# Access to interventional therapies for cancer pain: An exploratory survey of cancer pain experts

**DOI:** 10.1016/j.inpm.2025.100727

**Published:** 2026-01-02

**Authors:** Elizabeth Roux, Amitabh Gulati, Anuj Bhatia, David Hao

**Affiliations:** a Harvard Medical School, 25 Shattuck Street, Boston, MA, 02115, USA; b Division of Pain Medicine, Department of Anesthesiology and Critical Care, Memorial Sloan Kettering Cancer Center, New York City, NY, USA; c Department of Anesthesia and Pain Management and Institute of Health Policy Management and Evaluation, University of Toronto, University Health Network-Toronto Western Hospital and Women's College Hospital, Toronto, Ontario, Canada; d Department of Anesthesiology, Mass General Brigham, Harvard Medical School, Boston, MA, USA

**Keywords:** Cancer pain, Nerve blocks, Radiofrequency ablation, Pulsed radiofrequency, Intrathecal drug delivery system, Spinal cord stimulation, Peripheral nerve stimulation, Transcutaneous electrical nerve stimulation, Vertebral augmentation, Epidural steroid injection, Perineural steroid injection, Cryoablation, Cordotomy, Complementary and alternative therapies, Chemical neurolysis

## Abstract

**Background:**

The substantial health burdens and prevalence of cancer-related pain both during and after treatment underscore the need for expanded access to cancer pain specialists and therapeutic pain treatments. Despite growing demand, cancer pain specialists face substantial barriers to providing effective care.

**Objective:**

This exploratory study sought to characterize perspectives from a small group of international pain experts to examine patterns of utilization and perceived accessibility of interventional therapies across cancer types and clinical practice settings.

**Methods:**

An international, anonymous survey of cancer pain experts, identified via rigorous definition criteria, was conducted using the Qualtrics platform. The survey evaluated eight cancer-related pain categories: head and neck cancer, pleural and rib-based lung cancer, pancreatic cancer, pelvic cancer, lumbosacral spine cancer, extremity cancer, chemotherapy-induced peripheral neuropathy, and bone metastases. Respondents were asked to indicate which treatments they currently use for each type of cancer and which they would use if they had access to them.

**Results:**

Cancer pain experts reported limited access to procedures with greater complexity. Some procedures had substantial variability in use and accessibility, specifically SCS, ITDD, permanent PNS, and nucleus tractus cordotomy. Complementary and alternative therapies were desirable but largely unavailable, particularly in academic settings.

**Conclusion:**

Despite the growing need for cancer pain management, specialists continue to face substantial barriers to delivering effective care. This exploratory survey of cancer pain experts identified patterns of reported use and access limitations for therapies across cancer types and practice settings. These findings suggest a relationship between procedural complexity and access barriers, with utilization and availability shaped by institutional resources and practice settings.

## 1 Introduction

Pain is a symptom with significant negative effects on quality of life, functional capacity, and mental health [1]. Cancer-related pain most often arises as a direct consequence of the malignancy itself, but may also result from oncologic treatments or pre-existing chronic conditions [2]. The prevalence of cancer-related pain is substantial, estimated at 50.3 % during active oncologic treatment, 35.8 % after curative treatment, and 54.6 % in advanced, metastatic, or terminal stages of disease [3]. Alarmingly, approximately 25 % of cancer patients are inadequately treated for their pain [4]. These high rates of pain and undertreatment are increasingly concerning, especially given the rising incidence of cancer [[5], [6], [7]]. Collectively, these trends underscore the need for expanded access to cancer pain specialists and, per the National Comprehensive Cancer Network Adult Cancer Pain Guidelines, the role of interventional procedures as an integral component of multimodal cancer pain management when clinically indicated [8].

Despite growing demand, cancer pain specialists face persistent barriers at systemic, professional, and patient levels. At the system level, limited supportive healthcare policies and inadequate funding for specialized infrastructure impede the delivery of advanced interventional therapies [9,10], particularly in resource-constrained settings [11]. At the clinical level, access may be limited because these procedures often involve specialized training, dedicated equipment or resources, and referral pathways that ideally connect them to a broader multidisciplinary care framework. As interventions grow more technologically complex, they require specialized expertise often lacking in standard training pathways [[9], [10], [11]]. Fellowship education in cancer pain is often inconsistent, leading to variability in procedural offerings and clinician confidence [12,13]. Referral patterns further limit access. Medical oncologists and palliative care physicians may inadequately assess pain or be unaware of interventional options [[9], [10], [11],^14^,^15^]. Patient-level factors compound these challenges; individuals may underreport pain, or like their clinicians, be unaware of interventional treatments [9,11,15,16].

These multifactorial barriers contribute to substantial variability in access to interventional therapies, despite their potential to alleviate suffering and improve quality of life. Accordingly, this exploratory study sought to characterize perspectives from a small group of international cancer pain experts to identify potential areas for further investigation. Specifically, we examined reported patterns of utilization and perceived accessibility of interventional therapies across cancer types and clinical practice settings.

## 2 Methods

An international, anonymous survey of cancer pain experts was conducted in 2025 using the Qualtrics platform. A purposive sampling strategy was employed to ensure that the selected participants met a rigorous definition of “cancer pain expert.” Eligibility required meeting at least two of the following criteria; for each category, all listed criteria had to be satisfied for that category to count toward eligibility.

Clinical Experience:

- Board-certified or fellowship-trained in Pain Medicine, Palliative Care, Anesthesiology, Physical Medicine & Rehabilitation (PM&R), Neurosurgery, or Interventional Radiology.
- A minimum of five years of clinical experience managing cancer-related pain.

Institutional Affiliation:

- Actively practice at a National Cancer Institute (NCI)-designated cancer center, high-volume academic institution, or tertiary referral center with a dedicated cancer pain service.

Procedural Expertise:

- Experience performing or referring for interventional therapies such as intrathecal drug delivery systems (ITDDs), spinal cord stimulation (SCS), ablative procedures (radiofrequency ablation, cryoablation, neurolytic blocks), or regional anesthesia techniques.

Academic and Research Involvement:

- At least two peer-reviewed publications, book chapters, or national/international presentations on cancer pain management.
- Membership in professional societies with a focus on cancer pain.

Leadership and Education Contributions:

- Faculty member or director of an ACGME-accredited pain medicine or palliative care fellowship, given required exposure to cancer pain in fellowship training.
- Participation in guideline development, expert panels, or policy initiatives related to cancer pain.

Participants were identified through professional networks, national society directories, academic conference speaker lists, and peer recommendations. In selecting this group, we intentionally focused on individuals who met our predefined eligibility criteria and represented a range of practice settings, geographic regions, and training backgrounds. Our goal in this initial exploratory phase was to engage a cohort likely to complete the survey and provide a foundational dataset in an area where no prior structured inquiry exists. This approach allowed us to generate preliminary data to refine the survey domains and ensure that the survey instrument is feasible and well-constructed.

The survey evaluated eight cancer-related pain categories: head and neck cancer, pleural and rib-based lung cancer, pancreatic cancer, pelvic cancer, lumbosacral spine cancer, extremity cancer, chemotherapy-induced peripheral neuropathy, and bone metastases. Respondents were asked to indicate which treatments they currently use for each type of cancer and which they would use if they had access to them.

## 3 Results

### 3.1 Survey demographics

Of 25 cancer pain experts invited to participate, 17 (68 %) completed the survey in full. All respondents specialized in pain medicine with over half (53 %) trained in anesthesiology. Regarding experience, 41 % had been in practice for more than 15 years post-training, 47 % for 6–15 years, and 12 % for 0–5 years.

Geographically, 14 respondents practiced in North America and 3 in South America. Practice settings were split between academic institutions (47 %) and mixed academic-community models (53 %); no respondents worked exclusively in community or private practice settings. Clinically, 76 % reported treating more than 20 patients with cancer-related pain per month; one respondent treated 11–20, and three treated 5–10 patients monthly.

Demographically, 42 % of respondents identified as women. Regarding ethnicity, 47 % identified as Hispanic or Latino, followed by 29 % Asian, 12 % White, 6 % Native Hawaiian or Pacific Islander, and 6 % Other.

### 3.2 Head and neck cancer

Of the surveyed cancer pain experts, 82 % treated head and neck cancer pain (Fig. 1A). The most frequently reported therapies used were facial nerve blocks (93 %), trigeminal nerve blocks (86 %), radiofrequency ablation (71 %), and pulsed radiofrequency (64 %). Fewer than half of respondents reported using cryoablation (43 %), temporary or permanent peripheral nerve stimulation (29 %), transcutaneous electrical nerve stimulation (TENS) (21 %), or complementary/alternative practices such as yoga and meditation (21 %).

**Fig. 1A:**
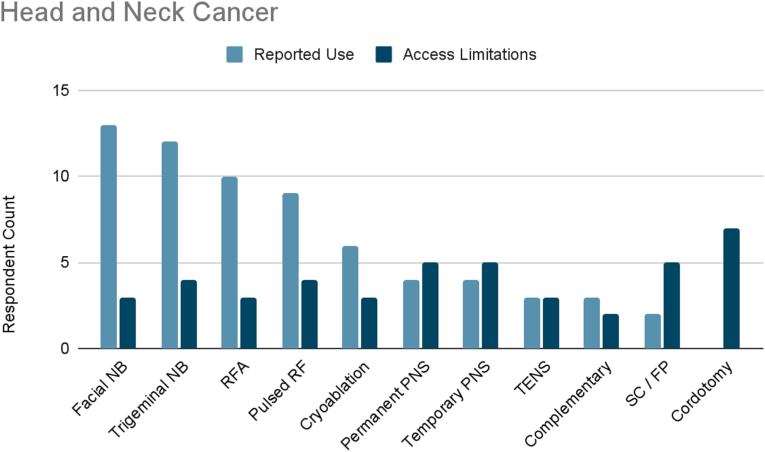
Head and Neck Cancer. Reported use and access limitations for therapies to treat head and neck cancer pain.

71 % of cancer pain experts who treated head and neck cancer pain identified therapies they would use if access were available. The most commonly cited therapies were nucleus tractus cordotomy (58 %), spinal cord and fascial plane stimulation (42 %), permanent peripheral nerve stimulation (42 %), trigeminal nerve blocks (33 %), and pulsed radiofrequency (33 %). Additionally, 25 % of respondents reported limited accessibility to TENS, temporary peripheral nerve stimulation, radiofrequency ablation, facial nerve blocks, cryoablation, or complementary/alternative practices.

### 3.3 Pleural and rib-based lung cancer

Of the surveyed cancer pain experts, 88 % treated pleural and rib-based lung cancer (Fig. 1B). The most frequently reported therapies used were epidural/perineural steroid injections (93 %), radiofrequency ablation (73 %), pulsed radiofrequency (73 %), cryoablation (67 %), and chemical neurolysis (67 %). About half (53 %) of clinicians reported use of SCS or ITDD. Less than half reported use of intrathecal neurolysis (40 %), complementary/alternative practices (40 %), TENS (27 %), permanent peripheral nerve stimulation (27 %), temporary peripheral nerve stimulation (13 %), and percutaneous cordotomy (7 %). Two respondents indicated use of “other” therapies: one used additional medications such as gabapentinoids, antidepressants, and NSAIDS, while the other reported using osteoplasty or kyphoplasty.

**Fig. 1B:**
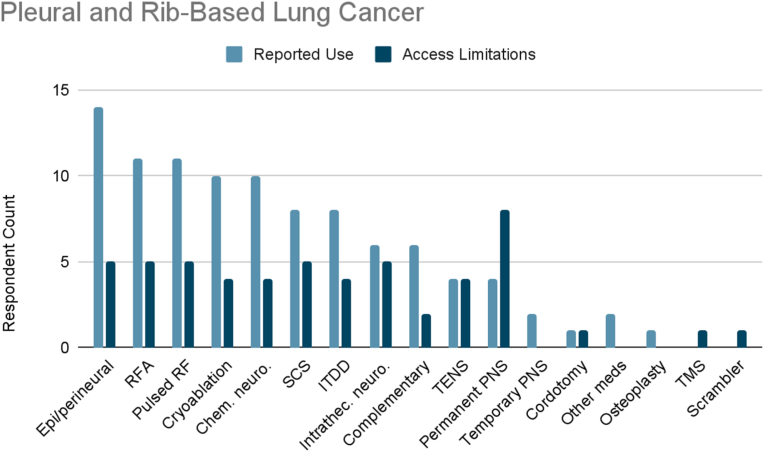
Pleural and Rib-Based Lung Cancer. Reported use and access limitations for therapies to treat pleural and rib-based lung cancer pain.

71 % of cancer pain experts who treated pleural and rib-based lung cancer pain identified therapies they would use if access were available. The most commonly cited therapy was permanent peripheral nerve stimulation (67 %). 42 % of respondents reported limited accessibility to SCS, radiofrequency ablation, pulsed radiofrequency, intrathecal neurolysis, or epidural/perineural steroid injections. 33 % of respondents indicated limited access to TENS, ITDD, cryoablation, or chemical neurolysis. Finally, limited accessibility was reported for complementary/alternative practices (17 %), percutaneous cordotomy (7 %), and “other” defined as transcranial magnetic stimulation (TMS) (7 %) and scrambler therapy (7 %).

### 3.4 Pancreatic cancer

Of the surveyed cancer pain experts, 88 % treated pancreatic cancer pain (Fig. 1C). The most frequently reported therapies used were splanchnic nerve blocks (100 %), celiac plexus blocks (73 %), ITDD (60 %), or radiofrequency ablation (53 %). Less frequently reported therapies used were complementary/alternative practices (27 %), pulsed radiofrequency (20 %), and cryoablation (7 %).

**Fig. 1C:**
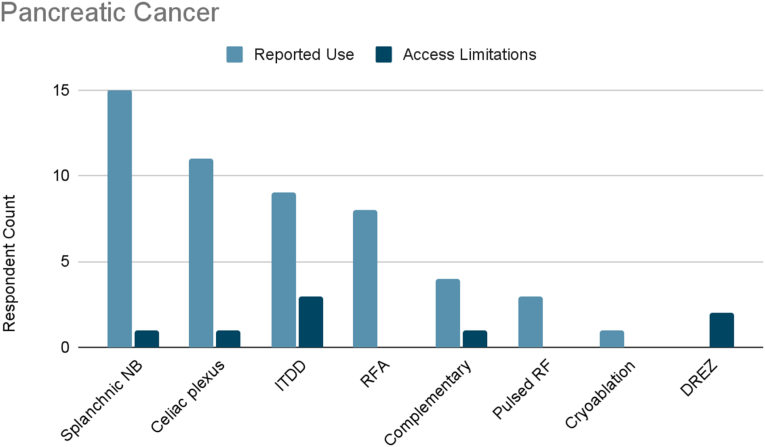
Pancreatic Cancer. Reported use and access limitations for therapies to treat pancreatic cancer pain.

58 % of cancer pain experts who treated pancreatic cancer pain identified therapies they would use if access were available. The most commonly cited therapy was ITDD (30 %). One fifth of respondents reported limited accessibility to neurosurgical procedures such as dorsal root entry zone (DREZ). Finally, 10 % of clinicians reported limited accessibility to splanchnic nerve block, celiac plexus block, or complementary/alternative practices.

### 3.5 Pelvic cancer

Of the surveyed cancer pain experts, 88 % treated pelvic cancer pain (Fig. 1D). The most frequently reported therapies used were hypogastric nerve blocks (100 %), ganglion impar blocks (87 %), and ITDD (60 %). Other reported therapies used included radiofrequency ablation (40 %), pulsed radiofrequency (33 %), complementary/alternative practices (33 %), SCS (20 %), cryoablation (20 %), and temporary peripheral nerve stimulation (7 %).

**Fig. 1D:**
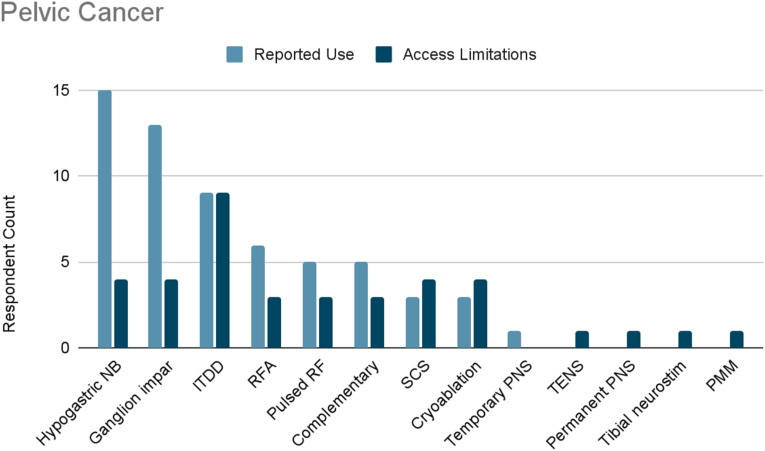
Pelvic Cancer. Reported use and access limitations for therapies to treat pelvic cancer pain.

65 % of cancer pain experts who treated pelvic cancer pain identified therapies they would use if access were available. The most commonly cited therapy in this category was ITDD (73 %). 36 % reported limited accessibility to hypogastric nerve block, ganglion impar block, SCS, and cryoablation. 27 % reported limited accessibility to radiofrequency ablation, pulsed radiofrequency, or complementary practices. Finally, limited accessibility was reported for TENS (9 %), permanent peripheral nerve stimulation (9 %), and “other” defined as tibial neurostimulation (9 %) and punctate midline myelotomy (9 %).

### 3.6 Lumbosacral spine cancer

Of the surveyed cancer pain experts, 82 % treated lumbosacral spine cancer pain (Fig. 1E). The most frequently reported therapies used were vertebral augmentation (100 %), radiofrequency ablation (79 %), and pulsed radiofrequency (64 %). Other reported therapies used included SCS (57 %), cryoablation (57 %), ITDD (50 %), permanent peripheral nerve stimulation (29 %), complementary/alternative practices (29 %), TENS (21 %), temporary peripheral nerve stimulation (21 %), and “other” defined as radiotherapy (7 %).

**Fig. 1E:**
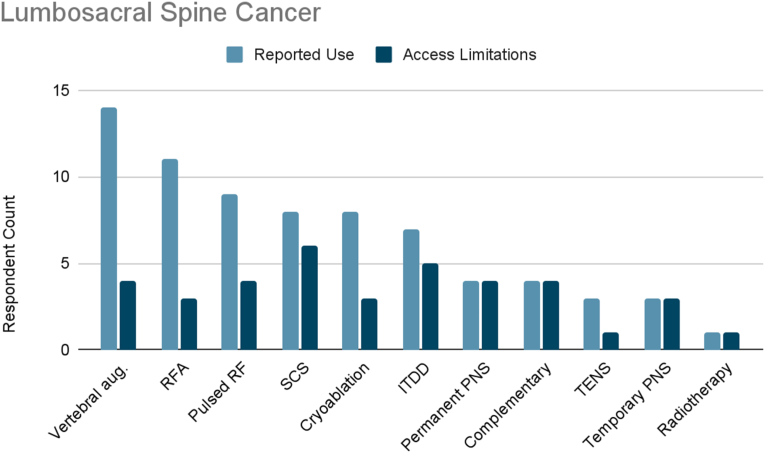
Lumbosacral Spine Cancer. Reported use and access limitations for therapies to treat lumbosacral spine cancer pain.

53 % of cancer pain experts who treated lumbosacral spine cancer pain identified therapies they would use if access were available. The most commonly cited therapies were SCS (67 %) and ITDD (56 %). 44 % reported limited accessibility to vertebral augmentation, pulsed radiofrequency, permanent peripheral nerve stimulation, or complementary/alternative practices. 33 % reported limited accessibility to temporary peripheral nerve stimulation, radiofrequency ablation, or cryoablation. Finally, 11 % reported limited accessibility to TENS or “other” defined as radiotherapy.

### 3.7 Extremity cancer

Of the surveyed cancer pain experts, 82 % treated extremity cancer pain (Fig. 1F). The most frequently reported therapies used were sympathetic blocks and chemoneurolysis (100 %), and most furthermore reported use of peripheral nerve blocks (79 %). About half of respondents reported use of SCS (57 %), pulsed radiofrequency (57 %), radiofrequency ablation (50 %), or ITDD (50 %). Other therapies used were permanent peripheral nerve stimulation (43 %), cryoablation (43 %), TENS (36 %), temporary peripheral nerve stimulation (36 %), complementary/alternative practices (36 %), percutaneous cordotomy (14 %), and “other” defined as botulinum toxin (7 %).

**Fig. 1F:**
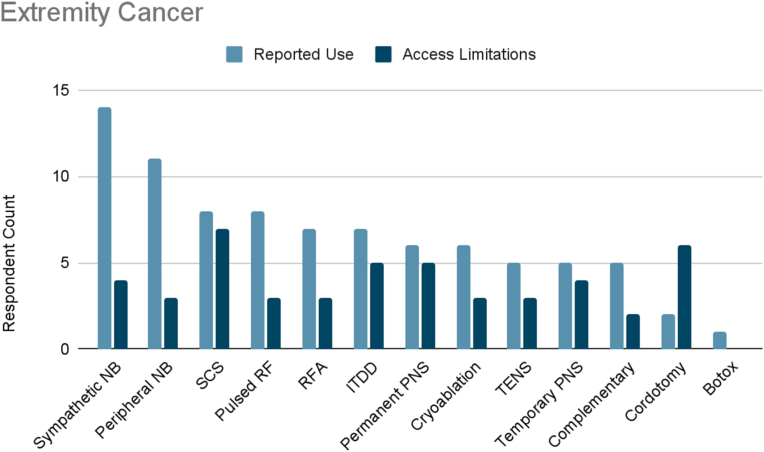
Extremity Cancer. Reported use and access limitations for therapies to treat extremity cancer pain.

65 % of cancer pain experts who treated extremity cancer pain identified therapies they would use if access were available. The most commonly cited therapies in this category were SCS (64 %), percutaneous cordotomy (55 %), permanent peripheral nerve stimulation (45 %), and ITDD (45 %). 36 % reported limited accessibility to temporary peripheral nerve stimulation and sympathetic blocks and chemoneurolysis. 27 % reported limited accessibility to TENS, radiofrequency ablation, pulsed radiofrequency, peripheral nerve blocks, or cryoablation. Finally, 18 % of respondents reported limited accessibility to complementary/alternative practices.

### 3.8 Chemotherapy-induced peripheral neuropathy

Of the surveyed cancer pain experts, 82 % treated chemotherapy-induced peripheral neuropathy (Fig. 1G). The most frequently reported therapies used were SCS (57 %), and pulsed radiofrequency (50 %). Other therapies used included ITDD (43 %), complementary/alternative practices (29 %), and cryoablation (14 %). 21 % reported use of TENS, temporary peripheral nerve stimulation, radiofrequency ablation, or permanent peripheral nerve stimulation. Finally, 36 % reported use of “other” therapies defined cumulatively as: antidepressants (18 %), anticonvulsants (18 %), lidocaine patch (12 %), botulinum toxin (6 %), and ketamine infusions (6 %).

**Fig. 1G:**
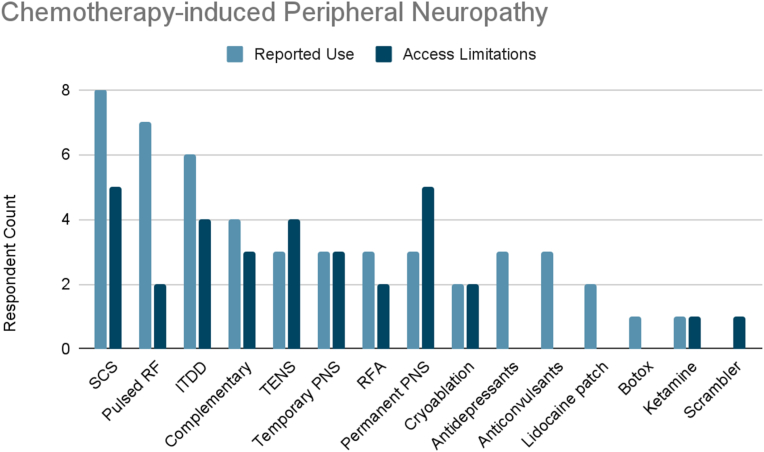
Chemotherapy-Induced Peripheral Neuropathy. Reported use and access limitations for therapies to treat chemotherapy-induced peripheral neuropathy.

59 % of cancer pain experts who treated chemotherapy-induced peripheral neuropathy identified therapies they would use if access were available. Half of these respondents cited limited accessibility to SCS and permanent peripheral nerve stimulation. Limited accessibility was also reported for TENS (40 %), ITDD (40 %), temporary peripheral nerve stimulation (30 %), complementary/alternative practices (30 %), radiofrequency ablation (20 %), pulsed radiofrequency (20 %), cryoablation (20 %), and “other” defined as ketamine (10 %) and scrambler therapy (10 %).

### 3.9 Bone metastases

Of the surveyed cancer pain experts, 82 % treated bone metastases pain (Fig. 1H). The most frequently reported therapies used were vertebral augmentation, epidural/perineural steroid injections (93 %), and radiofrequency ablation (79 %). Other therapies used included ITDD (57 %), pulsed radiofrequency (43 %), and cryoablation (43 %). 29 % reported use of SCS, complementary/alternative practices, or chemical neurolysis. 14 % reported use of TENS or permanent peripheral nerve stimulation. 7 % reported use of temporary peripheral nerve stimulation, “other” defined as steroid burst treatment, or “other” defined as erector spinae plane (ESP) block.

**Fig. 1H:**
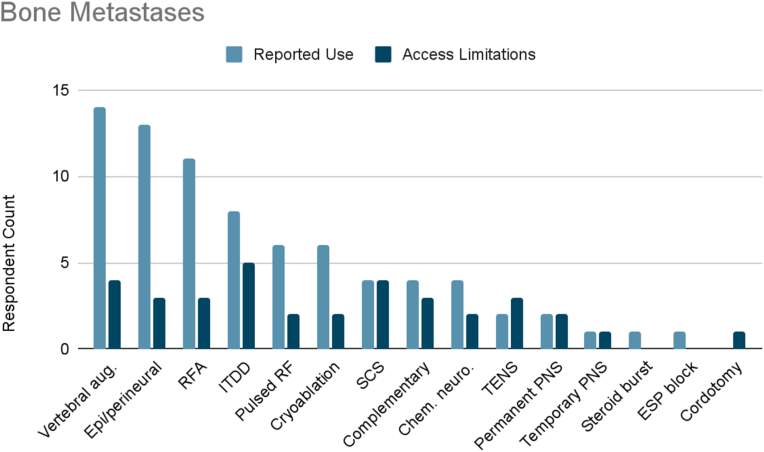
Reported use and access limitations for therapies to treat bone metastases pain.

47 % of cancer pain experts who treated bone metastases pain identified therapies they would use if access were available. The most commonly cited therapy in this category was ITDD (63 %). Half of respondents reported limited accessibility to vertebral augmentation or SCS. 38 % reported limited accessibility to TENS, radiofrequency ablation, epidural/perineural steroid injections, or complementary/alternative practices. One fourth of respondents reported limited accessibility to pulsed radiofrequency, permanent peripheral nerve stimulation, cryoablation, or chemical neurolysis. Finally, limited accessibility was reported for temporary peripheral nerve stimulation (13 %) and percutaneous cordotomy (13 %).

### 3.10 Trends across cancer types

Exploratory analysis across cancer types revealed distinct patterns in both the reported use of and limited accessibility to cancer pain therapies among respondents. For therapies that respondents reported they would use if available but currently lack access to, limited accessibility was categorized as significant (≥8 respondents), moderate (4–7 respondents), or mild (<4 respondents).

**Fig. 2:**
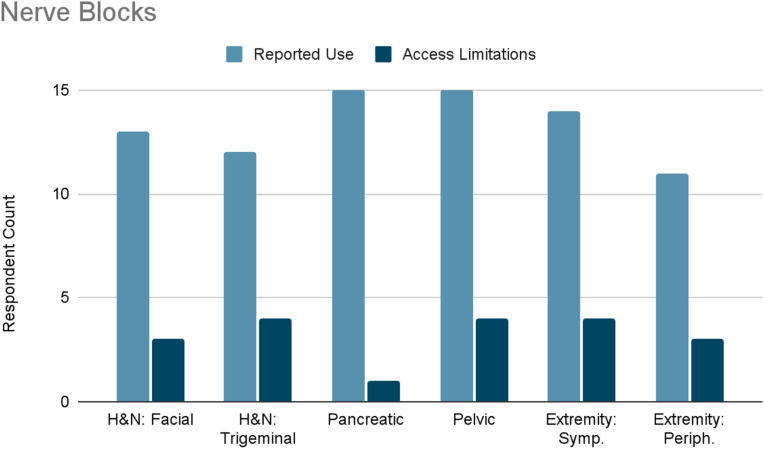
Reported use and access limitations of nerve blocks for the treatment of different types of cancer pain.

Nerve blocks demonstrated strong anatomical specificity in use patterns (Fig. 2). Splanchnic nerve blocks and celiac plexus blocks were most commonly used for pancreatic cancer pain, hypogastric and ganglion impar blocks for pelvic cancer pain, sympathetic and peripheral nerve blocks for extremity cancer pain, and facial/trigeminal nerve blocks for head and neck cancer. Access to these interventions was generally adequate, though mild to moderate limited accessibility was reported.

**Fig. 3:**
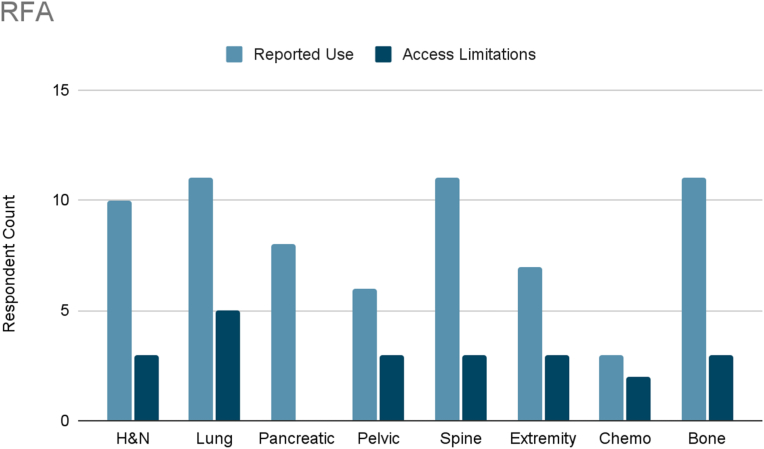
Reported use and access limitations of radiofrequency ablation for the treatment of different types of cancer pain.

**Fig. 4:**
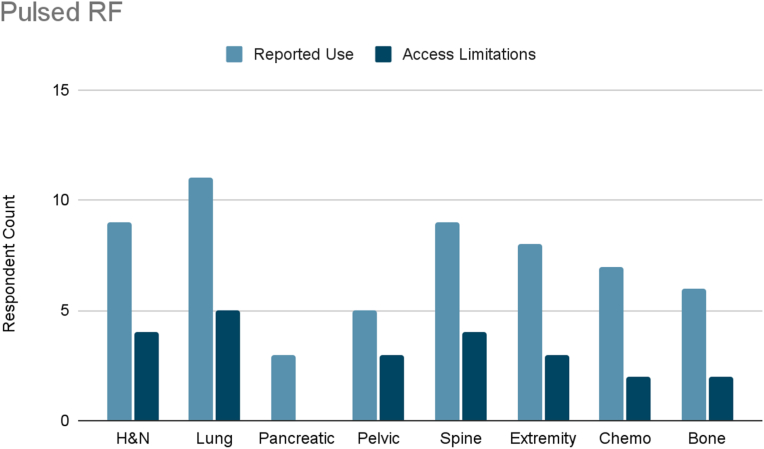
Reported use and access limitations of pulsed radiofrequency for the treatment of different types of cancer pain.

**Fig. 5:**
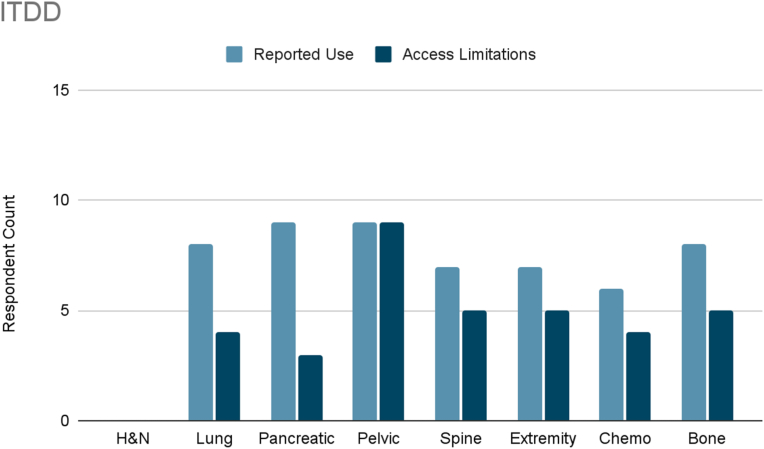
Reported use and access limitations of intrathecal drug delivery systems for the treatment of different types of cancer pain.

**Fig. 6:**
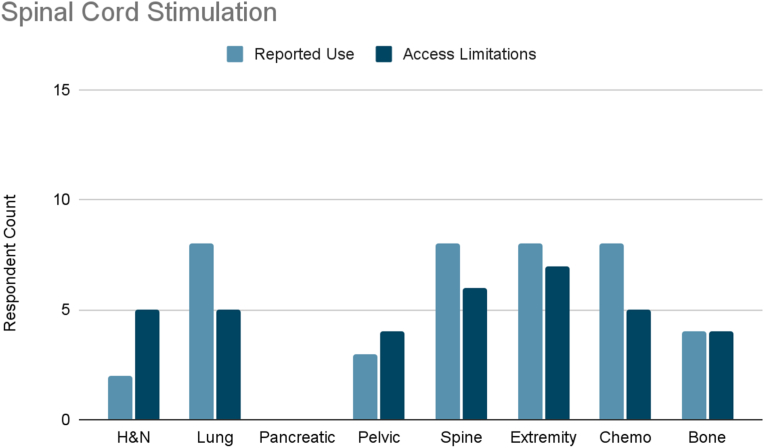
Reported use and access limitations of spinal cord stimulation for the treatment of different types of cancer pain.

**Fig. 7:**
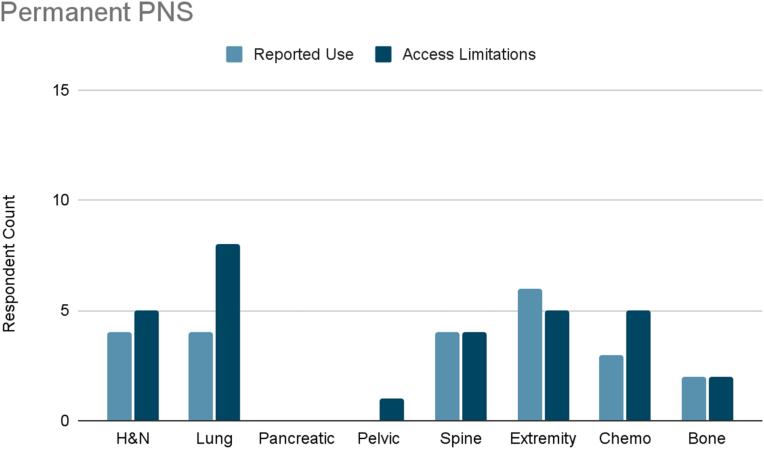
Reported use and access limitations of permanent peripheral nerve stimulation for the treatment of different types of cancer pain.

Several interventional therapies demonstrated consistent usage across cancer types. Radiofrequency ablation and pulsed radiofrequency were frequently used in six cancer types (head and neck, pleural/rib-based lung, lumbosacral spine, extremity, chemotherapy-induced neuropathy, and bone metastases), with mild to moderate limited accessibility reported (Fig. 3, Fig. 4). ITDD demonstrated broad applicability, particularly for pancreatic, pelvic, lumbosacral spine, extremity, chemotherapy-induced neuropathy, and bone metastases pain (Fig. 5). However, accessibility limitations were more pronounced, with significant restrictions reported for pancreatic cancer and moderate for most other cancer types. SCS was commonly used to treat lumbosacral spine pain, extremity pain, and chemotherapy-induced neuropathy, though access to SCS for these indications was reported to be mildly to moderately limited (Fig. 6). Respondents also cited limited accessibility of SCS for managing pain related to head and neck cancers, pelvic tumors, and bone metastases. Permanent peripheral nerve stimulation was reported as mildly to moderately used across all cancer types, yet moderate to significant limited accessibility was identified, particularly in head and neck, pleural and rib-based lung, lumbosacral spine, extremity, and chemotherapy-induced pain (Fig. 7).

**Fig. 8:**
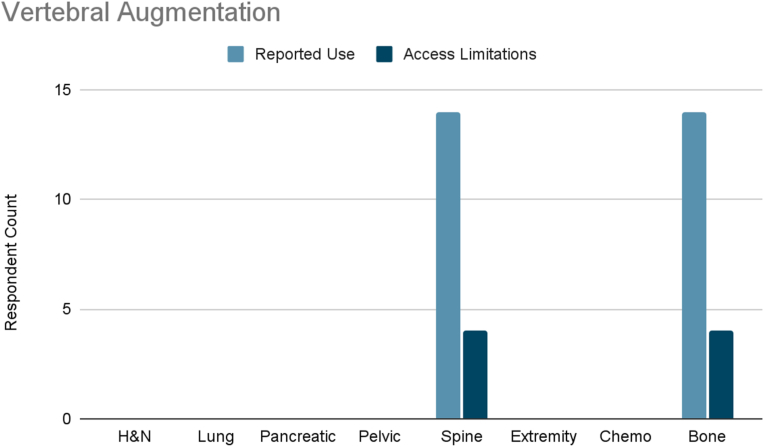
Reported use and access limitations of vertebral augmentation for the treatment of different types of cancer pain.

**Fig. 9:**
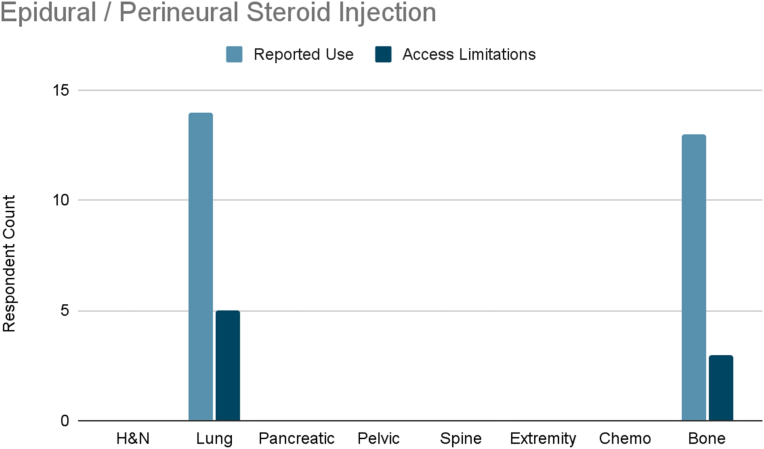
Reported use and access limitations of epidural and perineural steroid injections for the treatment of different types of cancer pain.

**Fig. 10:**
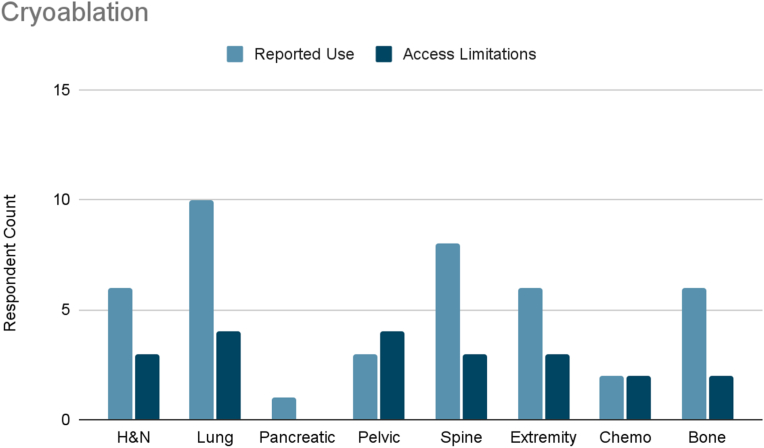
Reported use and access limitations of cryoablation for the treatment of different types of cancer pain.

**Fig. 11:**
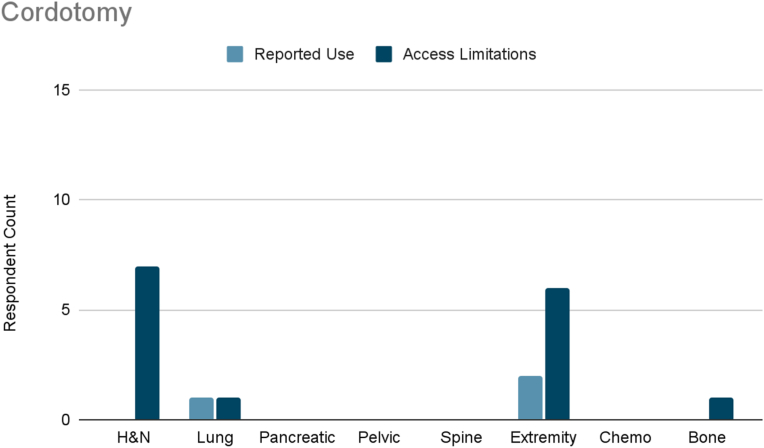
Reported use and access limitations of cordotomy for the treatment of different types of cancer pain.

**Fig. 12:**
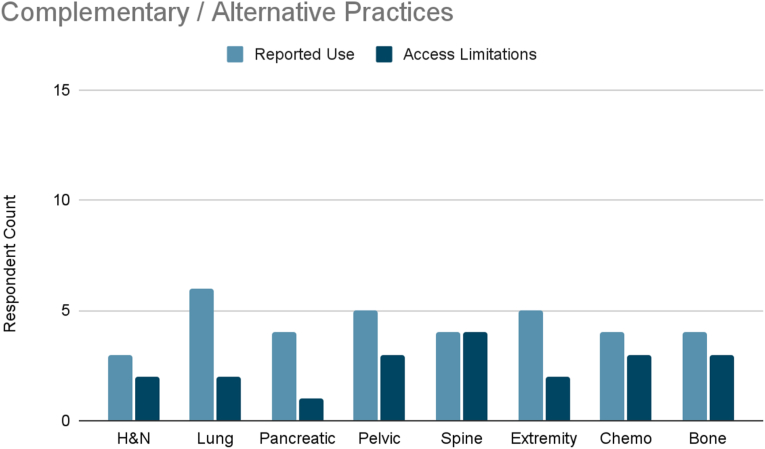
Reported use and access limitations of complementary and alternative practices for the treatment of different types of cancer pain.

Several interventions were more cancer-type specific. Vertebral augmentation was primarily used for lumbosacral spine and bone metastases pain (Fig. 8). Epidural or perineural steroid injections were the most used intervention for pleural and rib-based lung cancer pain and the second used for bone metastases (Fig. 9). Both showed moderate access limitations. Cryoablation ranked fifth and sixth in reported use for pleural and rib-based lung cancer pain and lumbosacral spine cancer pain, respectively, with mild to moderately limited accessibility (Fig. 10). Nucleus tract cordotomy and percutaneous cordotomy were rarely used but identified as interventions with moderate access limitations for head and neck cancer pain and extremity pain, respectively (Fig. 11). Complementary and alternative practices such as yoga and meditation were consistently reported across cancer types to have mild to moderate use and corresponding mild to moderate limited accessibility (Fig. 12).

### 3.11 Trends across practice settings

Exploratory analysis of reported use and access limitations revealed distinct differences between academic (n = 8) and mixed academic-community (n = 9) practice settings. Proportional use and access barriers were calculated by averaging reported frequencies across all cancer types, normalized by the number of respondents in each group (Fig. 13B, Fig. 13C, Fig. 13AA–C).

**Fig. 13A:**
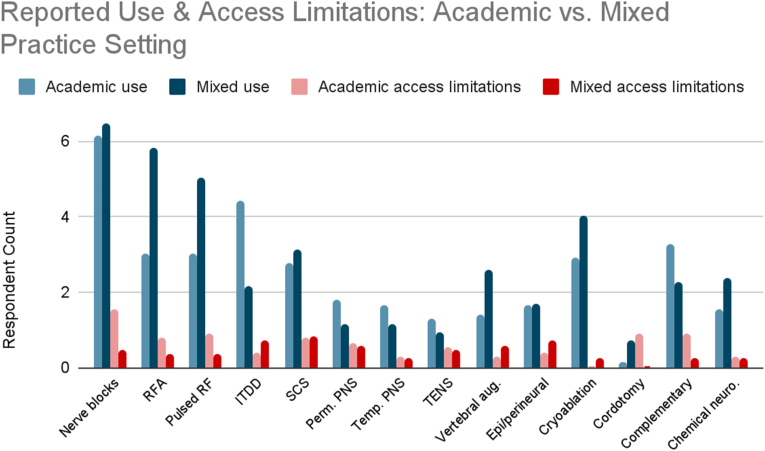
Reported use and access limitations for cancer pain therapies in academic and mixed practice settings.

**Fig. 13B:**
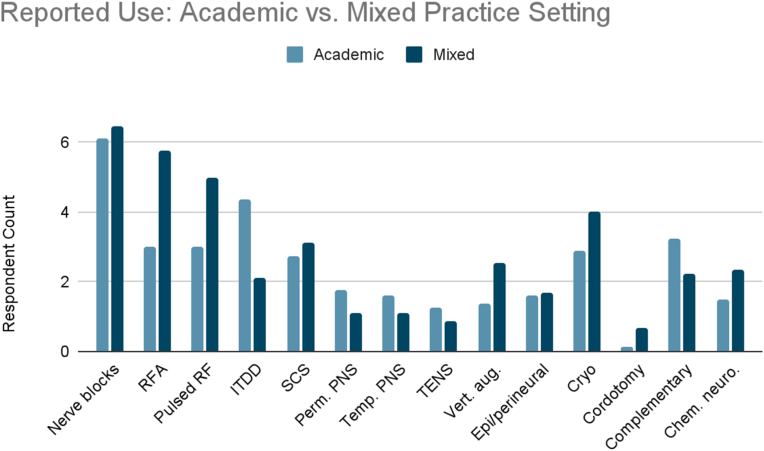
Reported use of cancer pain therapies in academic and mixed practice settings.

**Fig. 13C:**
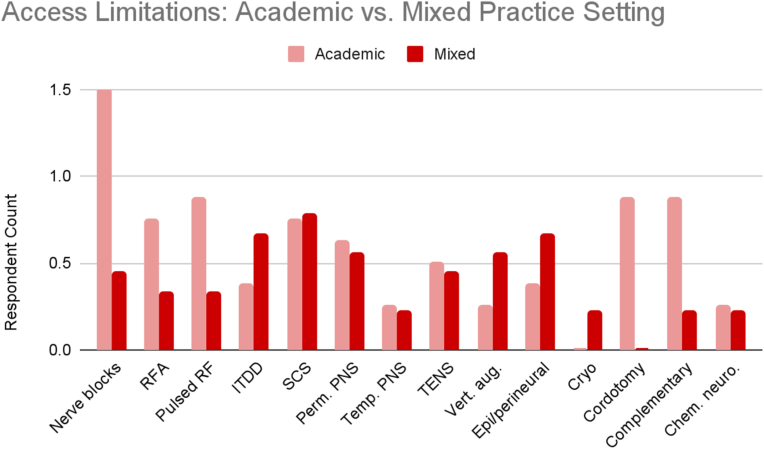
Access limitations for cancer pain therapies in academic and mixed practice settings.

Mixed practice settings reported higher use of ablative procedures. Radiofrequency ablation was used 1.8 times more frequently compared to academic settings, with pulsed radiofrequency showing a similar pattern. Other interventions more commonly used in mixed settings included vertebral augmentation and cryoablation. Academic practices reported greater use of advanced implantable and nonpharmacologic therapies. Use of ITDD was twice as high compared to mixed settings. Academic respondents also reported higher use of permanent peripheral nerve stimulation (1.3x), temporary peripheral nerve stimulation (1.2x), TENS (1.5x), and complementary and alternative therapies (1.4x).

Access comparisons revealed that academic respondents reported significantly more access limitations than those in mixed-practice settings. These included more than threefold greater reported barriers to nerve blocks, including facial and trigeminal nerve blocks for head and neck cancer pain, splanchnic and celiac plexus blocks for pancreatic cancer pain, hypogastric and ganglion impar blocks for pelvic cancer pain, and sympathetic and peripheral nerve blocks for extremity cancer pain. Similarly, threefold higher barriers were reported for cordotomy and complementary/alternative medicine. At least twofold greater barriers were noted for radiofrequency ablation and pulsed radiofrequency. In contrast, respondents in mixed settings reported greater difficulty accessing epidural and perineural steroid injections.

## 4 Discussion

This study suggests variability in how cancer pain therapies are utilized and accessed across practice settings. Despite guideline-supported roles for interventional therapies such as those presented by the National Comprehensive Cancer Network, respondents reported gaps in availability and uneven adoption across systems. The following sections provide context for these patterns and outline potential implications for clinical practice and system-level planning.

### 4.1 Interventional therapy access barriers

Exploratory analysis revealed a pattern linking procedural complexity with reported access limitations, with differences observed across practice settings. SCS, ITDD, permanent peripheral nerve stimulation, and nucleus tractus cordotomy were reported as frequently used by some clinicians yet unavailable to others, highlighting substantial variability in access. These interventions often require operating room infrastructure, specialized equipment and expertise, multidisciplinary coordination, and trial procedures, contributing to multiple potential barriers.

ITDD was used twice as often in academic settings but showed nearly double the reported access limitations in mixed practices, suggesting that infrastructure and system support may be key determinants of availability. Pancreatic cancer pain, in particular, demonstrated a 30 % rate of reported interest in ITDD use if access were available, representing a critical gap in care. SCS was reported as useful across multiple cancer types, particularly lumbosacral spine, extremity, and chemotherapy-induced pain, yet access remained mildly to moderately limited across both practice settings. Permanent peripheral nerve stimulation followed a similar pattern, with greater reported use in academic settings but consistently high unmet need across both settings. Nucleus tract cordotomy showed especially high unmet need for head and neck cancer, with access limitations reported three times more often in academic settings. Radiofrequency ablation and pulsed radiofrequency were used nearly twice as often in mixed practice settings, while academic respondents reported twice the rate of access barriers. Similarly, access to nerve blocks and percutaneous cordotomy was three times more limited in academic practices.

Some interventions demonstrated consistent use with only mild to moderate access limitations, indicating relatively adequate availability. These included vertebral augmentation for lumbosacral and bone metastatic pain, epidural and perineural steroid injections for pleural, rib, and bone metastases, and cryoablation. Use of these procedures was slightly more common in mixed practice settings.

### 4.2 Complementary and alternative medicine: an underutilized resources

Complementary and alternative practices, such as yoga and meditation, showed mild to moderate reported use across all cancer types, but were associated with notably greater access limitations in academic settings at a rate 3.4-fold higher than in mixed practices. While academic cancer and pain centers may have access to integrative medicine programs and trained practitioners [17], institutional barriers may limit their implementation. These may include limited administrative support, lack of reimbursement pathways, or burdensome approval process for new services. In contrast, community-based settings may face more patient-centered barriers such as out-of-pocket costs, lack of insurance coverage (e.g., Medicare exclusions), and lower socioeconomic status or health literacy [17,18]. Despite these challenges, the consistent interest in complementary and alternative therapies across practice settings, regardless of reported access, suggest untapped potential in broadening the scope of comprehensive cancer pain management.

## 5 Limitations

This study was exploratory in nature and relied on a convenience sample, which limits the generalizability of the findings. As the primary aim was to develop and preliminarily test a new survey instrument, the results should be interpreted as foundational rather than definitive.

Specifically, the limitations of this exploratory study are defined as follows. First, the sample size was relatively small, with only 17 of 25 invited experts completing the survey constituting a 68 % response rate. A larger sample might provide greater statistical power and more robust conclusions. Second, the geographic representation was limited, with respondents primarily from North America (14) and only 3 from South America. The absence of representation from other continents including Europe, Asia, Australia, and Africa limits the generalizability of findings to global cancer pain management practices. Third, the study lacked representation from purely community or private practice settings, as all respondents worked in either academic institutions or mixed academic-community models. Fourth, the purposive sampling strategy, while ensuring expertise among respondents, may have introduced selection bias. Fifth, the survey design relied on self-reported utilization patterns and perceived access limitations, which may be subject to recall bias and subjective interpretation. Objective measures of treatment availability, referral patterns, and patient outcomes were not assessed, limiting our ability to validate reported access barriers.

Despite these limitations, this study offers preliminary insights into the landscape of interventional cancer pain management and identifies key areas that warrant systematic investigation and policy attention. Future research should administer the refined instrument to a larger and more diverse population to evaluate its broader applicability, strengthen its properties, and further validate the patterns observed in this initial exploratory phase.

## 6 Conclusion

Despite the growing need for cancer pain management, specialists continue to face substantial barriers to delivering effective care. This exploratory survey of cancer pain experts identified patterns of reported use and access limitations for therapies across cancer types and practice settings. These findings suggest a relationship between procedural complexity and access barriers, with utilization and availability shaped by institutional resources and practice settings.

These observations underscore the need for further research to delineate the systemic, institutional, and patient-level barriers that contribute to differential access. Further work should expand the scale and geographic scope of respondents to capture a more representative international perspective, link self-reported patterns with objective utilization and reimbursement data, and incorporate policy analyses to identify structural determinants of inequity. Together, these efforts will guide strategies to expand equitable access to comprehensive, multimodal cancer pain care worldwide.

## Interests & disclosures

The authors have no competing interests or disclosures to declare.

## IRB statement

All procedures were performed in compliance with relevant laws and institutional guidelines of the Harvard Longwood Campus (HLC) Institutional Review Board. HLC institutional review board approval was waived through the use of the HLC IRB Decision Tool, which determined that this project does not meet the federal definitions requiring an IRB application.
